# American Medical Association—Committee on the Cure of Reducible Hernia

**Published:** 1851-12

**Authors:** 


*American Medical Association—Committee on the Radical Cure of Reducible Hernia.*

To the Members of the Medical Profession Throughout the United States.—The undersigned are a Committee of the American Medical Association, to report on "the radical cure of reducible hernia." They are desirous of obtaining from their professional brethren any information that is calculated to throw light on this important and interesting subject.

They therefore take the liberty of proposing the following questions. An answer to any or all of them, or any facts connected with the branch of surgery on which they are directed to report, would be gratefully received.

1st. Have you been in the practice of treating reducible hernia with a view to its radical cure?

2d. Have you ever performed any surgical operation for this purpose?

3d. If so, please to describe the operation and the mode of performing it.

4th. What proportion of cases, of all in which you have operated, has been cured?

5th. Have any alarming or fatal effects, in any instance, been caused by the operation?

6th. If so, please describe them.

As the Report must be made at the Annual Meeting of the Association, to be held in Richmond, Va., in May next, it is desirable that the answers to the above questions should be forwarded to any one of the Committee on or before March 1st, 1852.

Geo. Hayward,

J. Mason Warren, } *Committee.*

S. Parkman.

P. S.—Editors of Medical Journals and publishers of newspapers, throughout the United States, are respectfully requested to give the above an insertion in their respective Journals.

Boston, November 26th, 1851."

We hope that there will be a general response to the inquiries of this committee. An immense amount of valuable matter on this subject, that could be made of great use to the medical profession, is lying idle in the note books of physicians. Every physician who can furnish even one case in answer to the queries of the circular, should feel himself under obligations to his profession to respond to the call of the American Medical Association, made through its Committee.

A very important change has taken place in the management of Hernia, within the memory of the writer. In the ordinary management of reducible hernia, the former practice was devoted merely to restraining the passage of the bowel through the abdominal ring, and trusses were devoted to that object. Strangulated hernia was the only kind that usually came under the notice of the surgeon, and the operation then, after the failure of well-directed trusses, was a very formidable one, so formidable indeed, that efforts at reduction were often too much prolonged, to promise much benefit from the operation.

In 1836 or '7, as well as the writer can remember, a Mr. Stagner, of Madison county, Ky., a poor farmer, accidentally struck upon a discovery which has been of incalculable benefit. He had, what is called, a double hernia, and the rings had relaxed so much, that he found it difficult to procure a truss that would keep the bowels in their proper place. He was compelled to renew his instruments frequently, and they soon gave way. On one occasion, he was waiting at a country shop, while his plough was undergoing repair, and his trusses were yielding to the pressure of the bowels. He looked about, and found a couple of blocks of wood, which he whittled into a shape to suit a notion he had just formed, viz: that if these blocks were placed between the truss and the skin of the abdomen, he might thereby aid the objects of his truss. As soon as he placed the blocks, he found a sense of supporting relief, that he had never experienced before, and the comfort was so great, that he determined to continue to wear the blocks. In the course of three or four weeks persistence in this plan, he found, to his great joy that he was well, and that it was no longer necessary for him to wear trusses.

Such is the history, so far as memory can be relied upon, of a discovery that has made a very important improvement in the practice of surgery, and which has saved a great many valuable lives, and relieved numbers of persons of a most grievous annoyance.

Sometime after Mr. Stagner perfected his discovery, he applied to the writer to act as his agent in the use of the truss. The improved truss was made of steel, and the pad was a block of wood, the block slightly resembling the longitudinal section of an egg. As soon as the discovery was made known, a number of persons sought its benefits, and in the course of the past thirteen or fourteen years, the writer has used it on upwards of one hundred persons, and but one of the number failed to be cured. That one had omental hernia, and the truss will not cure that form of rupture. The object of Stagner's truss was to excite adhesive inflammation in the neighborhood of the rings, by what John Hunter called contiguity,—that is, by exciting inflammation in the skin and adjacent tissues, an inflammatory action was established about the ring, in such a way as to obliterate the canal through which the bowel was in the habit of passing.

In one case of double hernia, which was afterwards cured by Stagner's truss, the writer was summoned to the patient, and found him suffering with all the agonies of strangulated hernia. The difficulty of reduction was very great. The hot bath, nauseating doses of tartar emetic, bleeding, tobacco fomentations, tobacco enemas, &c., all failed to secure the reduction. A consultation was requested, and Dr. R. Wantyn was called in. After many efforts on his part, at reduction, he decided that the safety of the patient demanded the removal of the stricture, by an operation, and we prepared every thing for its performance. The patient was laid upon the table, and was so greatly alarmed by the sight of the case of instruments, that we succeeded in reducing the bowel during his fright. This patient was one of the first on whom Stagner's truss was placed, in Louisville, and he was effectually cured of his double rupture. He afterwards became afflicted with hydrocele, and the writer frequently operated upon him by tapping, but the calls for this, were so frequent, that he at length consented to undergo a more formidable operation, which was performed by Prof. J. B. Flint, greatly to the benefit of the patient. Whether this hydrocele was a consequence of the pressure of Stagner's truss, we are not prepared to say, for in numerous applications of it since that time, down to within the past few weeks, no such result has been seen in any other case. We are constantly in the habit of applying Stagner's truss, and always expect a cure from its proper and persistent use. Mr. T. Brown of this city, makes a superior truss, on Stagner's principle, and we always use it.

We never performed, but on one occasion, the operation for cutting the stricture in hernia. That was in 1835. The negro boy, the subject of the operation, resided 15 miles from this city, and we were summoned to his case about 9 o'clock, at night. The weather was extremely cold, and the roads so filled with snow, thet our travelling was prolonged to one o'clock in the night. The negro was perfectly cold, speechless, and pulseless, and all attempts at reduction proving fruitless, the master of the boy requested that the operation should be performed. This was declined on the ground that it could be of no service, and would add, unnecessarily, to the sufferings of the dying boy. This judgment was overruled by the joint importunities of the family physician, and the owner of the servant, both declaring that they saw the desperate condition of the case, and that they would not hold the operator accountable for a fatal result. In these circumstances the operation was performed. The parts were carefully dissected to the seat of the stricture, which was found to be in the internal ring. Upon cutting the stricture, the bowel was released, in a state of mortification, over a space of some seven or eight inches. The pulse soon returned, in a feeble degree, the boy recovered his speech, and the extremities began to show some warmth. Great hopes were entertained of the recovery of the boy, which were not shared in, however, by the operator, and after struggling a few hours, the boy died.

This is the sum of our experience in the matters connected with the inquiries of the committee. We have several patients now under treatment with Stagner's truss, and we may report them to the committee, before they make their report to the Association.

We have spoken of Stagner's truss only, because we consider, Chase's Hoods', and all others of their kind, as unimportant modifications of Stagner's discovery. We have had no reason to make any deviation from Stagner's principle. B.

